# The experiences of 585 people when they tried to withdraw from antipsychotic drugs

**DOI:** 10.1016/j.abrep.2022.100421

**Published:** 2022-03-17

**Authors:** John Read

**Affiliations:** School of Psychology University of East London London E15 4LZ, UK

## Abstract

**Introduction:**

Many recipients of antipsychotic drugs try to stop taking them, primarily because of distressing adverse effects. Little research has been undertaken into the withdrawal symptoms that ensue.

**Methods:**

In an online survey 585 antipsychotic users, from 29 countries, who had tried to stop taking the drugs, were asked specific questions about the process and the open question: ‘What were the effects of withdrawing from the medication?’ 44% had a diagnosis in the ‘schizophrenia’ spectrum.

**Results:**

Responding to specific questions, 72% reported classical withdrawal effects of the kind associated with other central nervous system medications, including nausea, tremors, anxiety, agitation and headaches. 52% of these categorized those effects as ‘severe’. 26% had tried four or more times to discontinue, and 23% took at least one year to successfully withdraw completely. In response to the open question, 73% reported one or more withdrawal effects, most frequently, insomnia, nervousness and extreme feelings; 26% reported one or more positive outcomes, most frequently more energy/alive and clearer thinking; and 18% reported psychosis.

**Conclusion:**

These findings are consistent with a small but growing body of literature on this topic. Prescribers need to inform themselves about the nature, frequency and intensity of withdrawal effects from APs, and about withdrawal psychosis. National guidelines, professional bodies’ statements, and drug company information urgently need to be updated to prevent the suffering that can occur when withdrawal is minimised, misunderstood or unsupported.

## Highlights

• 72% of an international sample of 585 antipsychotics users report withdrawal effects when they try to stop taking them.
• none recall being told anything about withdrawal effects, dependence, rebound psychosis, the need to reduce gradually, by the prescriber.
• 26% report positive effects of withdrawing, such as feeling more alive and more like themselves.

## 1 Introduction

Antipsychotic medications [APs] are commonly prescribed to adults diagnosed with ‘schizophrenia spectrum’ disorders. They are also increasingly prescribed, often in conjunction with other psychiatric drugs, to people with a range of other diagnoses, as well as to children, older people, and prisoners (Hutton et al., 2013, Larsen-Barr et al., 2018a). Only about half of the people prescribed antipsychotics in the UK primary care system have a diagnosis indicative of psychosis or bipolar disorder; other common diagnoses are anxiety, depression, dementia and sleep disorders (Marston, Nazareth, Petersen, Walters, & Osborn, 2014). There were 3.3 million prescriptions of APs in England in the third quarter of 2020/2021, an increase of 17% from 2015/2016 (NHS, 2021).

### 1.1 Adverse effects

APs have a range of serious adverse effects, in the following domains: neurological, metabolic, cognitive, affective, anticholinergic, autonomic, cutaneous, and hormonal (Hutton et al., 2013, Longden and Read, 2016, Moncrieff, 2015, Morrison et al., 2012, Murray et al., 2016). Specific adverse effects of particular concern include: tardive dyskinesia, sexual dysfunction, sedation, dizziness, akathisia, dry mouth, weight gain, reduced brain volume, and shortened life span (Ho et al., 2011, Hutton et al., 2013, Longden and Read, 2016, Weinmann and Aderhold, 2010). The most common adverse effects reported by 439 users of an Internet site were sedation, cognitive impairment, emotional flattening and loss of interest (Moncrieff, Cohen & Mason, 2009). The largest online survey of AP users to date (which forms the dataset used in this paper) found an average of 11 adverse effects, most frequently ‘drowsiness, feeling tired, sedation’ (92%), ‘loss of motivation’ (86%), ‘slowed thoughts’ (86%), and ‘emotional numbing’ (85%). Suicidality as a result of the APs was reported by 58% of respondents (Read & Williams, 2019).

### 1.2 Discontinuing

These adverse effects are a major factor in the high rates of people attempting to discontinue their APs (Cooper et al., 2005, Read and Williams, 2019). It is estimated that about three quarters stop the drugs within 18 months (Lieberman et al., 2005). People frequently make independent changes to their AP medication regimes in attempts to manage the adverse effects (Bülow, Andersson, Denhov, & Topor, 2016). Reviews of ‘nonadherence’ to APs have found averages of about one in four (Nosé, Barbui & Tansella, 2003) and about a half (Lacro, Dunn, Dolder & Jeste, 2002).

Most (70%) of respondents to ‘*The Experiences of Antidepressant and Antipsychotic Medication Survey’* (Read & Williams, 2018), the questionnaire used for the current paper, had tried to stop taking the drugs. The most common reasons people wanted to stop were the side effects (64%) and worries about long-term physical health (52%) (Read & Williams, 2019).

### 1.3 Withdrawal

#### 1.3.1 Classic withdrawal symptoms

The withdrawal effects of APs have been largely ignored for decades. Any negative effects of reducing or coming off completely have traditionally been interpreted as a return of the condition for which the drugs were prescribed, leading to urgent reinstatement of the drugs. This parallels the minimisation and misdiagnosing of the withdrawal effects of antidepressants, which only very recently have been recognised to be very common and frequently severe and long-lasting (Davies and Read, 2019, Framer, 2021, Horowitz and Taylor, 2019, Lewis, 2021, Taylor et al., 2019, White et al., 2021).

A review (Chouinard et al., 2017) found that APs share a range of ‘classic symptoms of withdrawal’ with all central nervous system drugs. These reactions, which usually emerge within a few days of stopping, include: nausea, tremors, anxiety, agitation, headaches, irritability, aggression, sleep disturbances and decreased concentration. There are, however, very few studies of the incidence, severity or duration of these classic withdrawal reactions following discontinuation of APs. The survey that generated the data for the current paper on 585 people who had tried to stop taking their APs, had found that 65% of the total number of 832 AP users responding to the survey had reported withdrawal effects (Read & Williams, 2019); and that half of those people (51%) described their withdrawal effects as ‘severe’. The presence of withdrawal effects was strongly correlated with duration of treatment. A smaller study found that 65 of 105 (62%) people who had attempted discontinuation reported unwanted withdrawal effects (Larsen-Barr, Seymour, Read & Gibson, 2018b). A recent review, which could find only five studies, calculated a weighted average of 53% individuals showing withdrawal symptoms after abrupt antipsychotic discontinuation (Brandt et al., 2020).

The larger survey also asked people to describe their overall experience of APs in their own words. One of the main negative experiences of taking APs was difficulty withdrawing from these drugs, with respondents stating, for example: “Withdrawal from the anti-psychotic was torturous and took a very long time. I would never choose to take them again, ever”; “Withdrawal symptoms were always blamed on relapse of my ‘disease’”; “I suffered hallucinations, and headaches during withdrawal even from stopping a low dosage” (Read & Sacia, 2020).

#### 1.3.2 Antipsychotic induced psychosis

APs blockade the dopamine system, and the brain tries to compensate for the blockade (Moncrieff, 2015). Nearly 50 years ago Dr Solomon Snyder, Professor of Psychiatry and Pharmacology at John Hopkins University had warned that:.

Something within the neurons recognises this sudden absence of neurotransmitter molecules at their appropriate receptor site and one way or another transmits a message back to the dopamine neurons saying something like the following: ‘We don’t have enough dopamine. Please send us some more!’ Whereupon the dopamine neuron in question proceeds to fire at a more rapid rate. (Snyder, 1974)

The brain’s attempted compensation involves an increase in the number, and sensitivity, of dopamine receptor cells (Chouinard et al., 2017). When an antipsychotic, and thereby the dopamine blockade, are removed, or reduced, the brain is overwhelmed with dopamine, partly because of the abnormal drug-induced sensitivity and number of dopamine receptor cells. This can result in a withdrawal psychosis, which is often mistaken for a return of the ‘schizophrenia’ that the drugs were intended to treat. This in turn often leads to a reinstatement of the drugs that have, paradoxically, caused the neurotransmitter abnormalities (Hutton et al., 2013). A 2006 reviewer of the available evidence concluded:.

There is evidence to suggest that the process of discontinuation of some antipsychotic drugs may precipitate the new onset or relapse of psychotic symptoms. Whereas psychotic deterioration following withdrawal of antipsychotic drugs has traditionally been taken as evidence of the chronicity of the underlying condition, this evidence suggests that some recurrent episodes of psychosis may be iatrogenic (Moncrieff, 2006)

There have been two recent, comprehensive reviews of the research literature on what now tends to be called ‘antipsychotic-induced Dopamine Supersensitivity Psychosis’ or ‘Supersensitivity Psychosis’ (Chouinard et al., 2017, Yin et al., 2017). Other terms are ‘Rebound Psychosis’, or ‘Withdrawal Psychosis’. Few studies have addressed the incidence or duration of withdrawal induced psychosis. Reported incidence ranges from 22% to 72% (Read, Davies, Montagu, Spada & Frederick, 2019). There is some evidence that antipsychotics with shorter half-lives (e.g. clozapine, metroclopromide, sulpiride, amisulpiride) are more likely to provoke the phenomenon (Chouinard et al., 2017, Moncrieff, 2006).

#### 1.3.3 Tardive dyskinesia

Tardive Dyskinesia (TD) is a disabling, often irreversible neurological disorder caused by APs. It involves uncontrollable movements of the face, tongue, arms and legs. The average prevalence of TD in people taking antipsychotics is about 30%, rising to 57% after 15 years of treatment with first generation antipsychotics. People over 50 are three to five times more likely than younger people to develop TD (D’Abreu et al., 2018, Hutton et al., 2013). The prevalence was thought to be lower for second generation, ‘atypical’, antipsychotics, but the difference has found to be slight or non-existent, or the consequence of second-generation antipsychotics being prescribed at lower dosages. It is listed here as a withdrawal effect because the symptoms of TD are often unmasked by the withdrawal of antipsychotics. Thus, the overt physical symptoms of TD are often either seen for the first time, or are exacerbated, after discontinuation, reduction or switching of antipsychotics.

### 1.4 Aims of the study

The primary aim of this study is to simply document the self-reported experiences of a large international sample of people who tried to come off APs. The correlates and duration of withdrawal symptoms are analysed. The paper also reports how many people were told about withdrawal effects when first prescribed the drugs.

## 2 Methods

This study was approved by the Human Research Ethics Committee of Swinburne University of Technology in Melbourne, Australia, where the data collection took place.

### 2.1 Instrument

‘*The Experiences of Antidepressant and Antipsychotic Medication Survey’* (Read & Williams, 2018) was based on the New Zealand *‘Views on Antidepressants’* questionnaire (Read et al., 2014, Read et al., 2018). Questions about antipsychotics were added, based on the relevant research (Read and Sacia, 2020, Read and Williams, 2019). The questionnaire was offered online and used Qualtrics survey software. It generated qualitative data (from open-ended questions) and quantitative data (from yes/no and multiple-choice questions), about the prescribing process, the effects of medications, causal beliefs about psychosis/depression, alternative treatments, and experiences of withdrawing from the medications.

This paper reports the data about withdrawing from antipsychotics generated by quantitative questions about how often and how quickly people tried to withdraw, and by the open question: ‘What were the effects of withdrawing from the medication?’ It also asked whehter people were told about withdrawal when prescribed the drugs.

### 2.2 Participants

Twelve per cent of participants were recruited via an online research company (GMI Research) and the remaining 78% via advertisements on social media and snowball sampling. Of the 2346 people who responded, 1067 reported that they had taken APs. However, 104 of these failed to tick ‘Yes’ to confirm that they met the following three inclusion criteria: ‘I have been taking or have previously taken antipsychotic medication continuously for at least one month’; ‘I am aged 18 or older’; and ‘I am not currently compulsorily detained in a psychiatric hospital’. Among the remaining 963 responses, 51 emanated from the same Internet Protocol (IP) address as another response, indicating use of the same computer. Of these 51, 23 were deemed a repeat response by the same person (based on identical demographics or similar responses). Of the remaining 938, 27 responded to ‘What is the name of your current or most recent antipsychotic medication?’ with a drug that is not an antipsychotic. Of the remaining 911, 79 completed only the demographics section, leaving 832 (Read & williams, 2019).

Of these respondents, 585 (70.3%) had tried to stop taking their antipsychotics at least once and were therefore eligible for inclusion in the current analyses.

### 2.3 Data analysis

Chi Squares were used to analyse relationships between categorical variables. Continuous variables were analysed using Spearman Rank correlation coefficients or two-tailed t-tests. The 319 people reporting withdrawal effects were classified as ‘severe’ cases if they used degree adverbs such as ‘extreme’, ‘intense’, ‘severe’ etc. to describe an effect, or if the symptoms lasted more than a year, or if five or more symptoms were listed.

## 3 Results

### 3.1 Sample characteristics

Of the 585 people eligible for inclusion, 71.0% were women. Ages ranged from 18 to 76 and averaged 42.8 years (SD 13.1). Respondents were from 29 countries, mostly the USA (26.0%), Australia (24.3%) and the UK (20.7%). Other countries contributing more than 1% were New Zealand (4.6%), Canada (3.8%), Netherlands (3.1%), Germany (2.6%), Ireland (2.6%), Denmark (2.6%), Norway (1.7%) and South Africa (1.2%). Countries providing from 1 to 5 participants were: Austria, Belgium, Croatia, Estonia, Finland, France, Greece, Iceland, India, Israel, Italy, Lithuania, Poland, Portugal, Romania, Spain, Sweden, Switzerland and Ukraine. The most frequently reported ethnicities (self-defined) were ‘white’/’Caucasian’ (48.1%), ‘Australian’ (10.4%) and ‘European’ (6.6%).

Approximately a quarter (26.3%) had taken antipsychotics for 1–12 months, 17.5% for 1–3 years, and 56.2% for more than three years. The 504 who named their ‘current or most recent antipsychotic medication’ cited 23 antipsychotics, most frequently quetiapine (34.7%), olanzapine (16.9%), aripiprazole (12.3%) and risperidone (11.7%). Analyses were conducted only on these four drug types. The majority reported a second generation (‘atypical) drug (91.3%), and that the drug was taken in pill form (96.2%) rather than by injection.

DSM-V groupings cited as ‘primary diagnosis’ by 3% or more of participants were: ‘Schizophrenia Spectrum and Other Psychotic Disorders’ – 37.1%; ‘Bipolar and Related Disorders’ − 21.2%; ‘Depressive Disorders’ − 21.0%; ‘Personality Disorders’ − 6.9%; and ‘Trauma and Stressor-Related Disorders’ − 3.1%. Secondary diagnoses included 6.9% in the schizophrenia spectrum, bringing the total (primary or secondary) for that group to 44.0%.

In the survey’s outcomes section (Read & Williams, 2019) more of the 585 respondents found that the drugs had ‘reduced the problems for which they were prescribed’ (52.2%) than thought they had been made ‘worse’ (32.0%). However, more reported that the drugs were in general ‘unhelpful’ (49.6%) than reported they were ‘helpful’ (35.3%). Far more reported that their ‘Quality of Life’ had been made worse (61.6%) than thought it had been ‘improved’ (28.6%).

### 3.2 Withdrawal numbers

A previous paper reported that 65.2% of the total sample of 832 reported withdrawal effects (Read & Williams, 2019); but this included respondents who had never tried to come off. Of the 565 who *had* tried to come off, and who answered the question about withdrawal effects, 410 (72.5%) reported some degree of withdrawal effects. Of these 410 respondents, 77 (18.8%) described the effects as ‘mild, 118 (28.8%) as ‘moderate’, and 215 (52.4%) as ‘severe’.

### 3.3 Correlates of withdrawal

As was the case for the total sample of 832 (Read & Williams, 2019), withdrawal effects were unrelated to gender, education or income. Those who reported withdrawal symptoms were, however, significantly older (43.6 years) than those who did not (40.8) (t = 2.23, df 559, p <.05). Duration of treatment was, again, significantly related to withdrawal symptoms (*X*^2^ = 22.53, p <.001). Of those who had taken the drugs for more than three years 79.3% reported some withdrawal effects, and 48.6% reported ‘severe’ withdrawal effects. Of those who had been on them for less than six months 60.9% reported some withdrawal effects, with 20.7% rating them as ‘severe’.

Abilify was related to a higher rate of withdrawal symptoms (81.7%) than Risperidone (60.7%; *X*^2^ = 6.25, p <.05) and Seroquel (67.6%; *X*^2^ = 4.26, p <.05). Olanzapine (74.7%) was not significantly different from the other three drug types. Furthermore, Abilify elicited significantly higher reports of severity than Risperidone (p <.01), as did Olanzapine (p <.05).

For those who had successfully withdrawn, the length of time taken to do so was positively related to experiencing withdrawal effects (*X*^2^ = 25.43, p <.001). Of those who stopped ‘cold turkey’ 61.4% experienced some withdrawal effects, with 26.7% reporting ‘severe’. Of those who took a year or more to withdraw, 93.3% experienced some withdrawal effects, with 70.7% reporting them as ‘severe’.

A positive relationship with the prescriber was negatively related to withdrawal symptoms (*X*^2^ = 16.00, p <.01). 61.5% of participants reporting a ‘very good’ relationship (on a five-point scale) experienced withdrawal symptoms, but 84.5% of those reporting ‘not at all good’ experienced withdrawal symptomso.

All three outcome measures (see Sample Characteristics) were strongly related to withdrawal effects, all at the p <.001 level. For example, 48.4% of those whose Quality of Life was ‘greatly improved’ by the drugs reported withdrawal symptoms, compared to 87.0% of those reporting that their Quality of Life was ‘a lot worse’ (*X*^2^ = 60.83, p <.001).

### 3.4 Number of attempts to withdraw

Of the 533 who had tried to stop and stated how many times they had done so, 214 (40.2%) had tried just once, 182 (34.1%) had tried two or three times, 103 (19.3%) had tried four to nine times, and 34 (6.4%) had tried 10 times of more.

### 3.5 Length of withdrawal process

Of the 526 who responded to ‘How did you go about withdrawing from anti-psychotic medication?’ 295 (56.1%) ticked ‘I slowly reduced my dose over a period of time before stopping entirely, 206 (39.2%) endorsed ‘I stopped abruptly all in one go’, and 25 (4.8%) had used both approaches.

Of the 268 who responded to ‘Approximately how long did it take you to reduce to no medication?’ 93 (34.7%) said 0–1 days, and 64 (23.0%) took a year or more. The other responses are presented in Table 1.

**Table 1:** Length of time to withdraw from medication (n = 268).

0–1 days	93 (34.7%)
2–7 days	17 (6.3%)
1–4 weeks	18 (6.7%)
1–2 months	33 (12.3%)
3–12 months	43 (16.0%)
1–2 years	38 (14.2%)
3–5 years	16 (6.0%)
6–10 years	5 (1.9%)
greater than10 years	5 (1.9%)

Length of withdrawal was positively correlated with duration of treatment (rho = 0.22, p <.001).

### 3.6 Effects of withdrawing

Five hundred and thirty-five people responded to the open question ‘What were the effects of withdrawing from the medication?’ Of these, 67 stated there were no effects (with four adding that this was because they withdrew slowly and two stating it was because they had only been on the drugs for a few weeks). Twenty-six said they could not recall whether there were any effects. Eight had never tried to come off. This left 434 responses citing one or more negative or positive effects.

The majority (319, 73.5%) reported one or more withdrawal effects (see Table 2, Table 3); 111 (25.6%) reported one or more positive outcomes (Table 4, Table 5), and 80 (18.4%) reported psychosis (Table 6, Table 7). Thirty-seven (8.5%) reported both withdrawal effects and psychosis; 34 (7.8%) reported both withdrawal and positive effects; and 5 (1.5%) reported both psychosis and positive effects. Table 8 gives examples of these mixed responses.

**Table 2:** Types of withdrawal effects reported by ten or more respondents.

Insomnia/sleeplessness	125	
Anxiety/nervousness	83	
Extreme/labile feelings	60	extreme emotionality 23, mood swings 21, mania/euphoria 16
Cognitive problems	40	confusion 12, concentration 11, memory 10, thinking 7
Nausea/vomiting	38	
Akathisia/restlessness/agitation	36	agitation 18, restlessness 11, akathisia 10
Suicidal thoughts/urges	32	
Aches/pains/cramps	32	
Dizziness/fainting	30	
Weight loss/low appetite	29	
Depression	28	
Headaches	26	
Tremors/shaking	21	
Sweating	20	including 3-night sweats
Irritable	18	
Agitation	17	
Anger/rage/aggression	15	
Crying	15	often extreme and/or unexplained
Sensitivity to light/sound	14	
Heart palpitations	14	
Brain ‘zaps’	12	
Vivid/bizarre dreams	11	
Tiredness/exhaustion	11

**Table 3:** Examples of the 130 cases of severe withdrawal effects.

Symptoms	Age, Gender, Country
2 days and nights of awful sweats and feeling very anxious	45, M, UK
complete insomnia and crushing anxiety for a month	51, F, UK
Insomnia, extreme headache, extremely high blood pressure, nausea, dizziness, agitation, suicidal ideation	59, F, USA
Nauseous, tummy aches, excruciating headaches, insomnia, restlessness, agitation, bed ridden	34, F, Australia
all kinds of severe things, from insomnia to anxiety, panic attacks, sweating, heart racing, nervousness	35, F, Austria
Worst anxiety I had experienced in my life - like a full-blown panic attack but spread out over several days (I don't think I actually wanted to die any other time in my life but then), no appetite, insomnia, being irritable and maybe slightly paranoid (because I was so afraid if I didn't “handle” it well, I would be forced to go back on them)	24,?, USA
No sleep, extreme fear and anxiety, extreme emotional instability, feeling like I was existing in a state between wake and sleep, depression, suicidal thoughts, roller coaster from hell	61, F, Australia
No sleep, extreme anxiety, dizziness, heart palpitations, resurfacing of trauma, memories of trauma and my life that the drugs disconnected me from came back, vertigo, stabbing pain in my head, chest pain, absolute terror, surges of emotion	40, F, Australia
Dizziness, brain fog, neuropathy, bed ridden, loss of weight, tinitus, chronic centralized pain, vision problems, coordination problems, processing problems, memory issues, anger, anxiety, depression	52, M, USA
With each reduction in dose you feel slightly destabilised and 'shaky' and have to process the trauma you have been through, as your emotions are no longer numb. So many of the symptoms that arise are like PTSD	38, F, Australia
For about two weeks, I became extremely depressed, anxious and agitated, and attempted suicide, but after about two weeks, I felt significantly less depressed and generally, back to my old self	26, F, Australia
Emotional outbursts, headaches, nausea, feeling terrified, feeling suicidal	22, F, Netherlands
I am in withdrawal now since 4 years!!! It is hell on earth!	?, F, Germany
Worse than any form of torture you could imagine….…Intractable insomnia, extreme intense anxiety, akathisia - an internal feeling of horror indescribable in words, wanting to commit suicide just to end the internal torment	35, M, Australia
Brain zaps, tingling. Light sensitivity, “like your brain is loose in your skull and slushing around”, crying 20x a day, Post-acute withdrawal: Intense apathy, zero motivation, anxiety, akathisia and depression unlike anything I'd ever experienced before. Lasted 2 years	28, F, Denmark
Brain zaps. Very very tired all the time	49, F, Ireland
Sleep deprivation. Severe concentration problems. Mood swings. Aggression. All since passed	40, M, Australia
Sweating, shaking, not being able to sleep all night, dreadful depression, only able to lie in bed and cry	45, F, UK
Aches and pains, nausea, extreme dizziness, slurred speach, high temperature, vomiting I thought I was going to die I felt soooo bad	63, F, South Africa
fever, heartpounding, the nerves in my body was kind of electric, I had thoughts of killing myself, its the most awful thing in my life	25, M, Denmark

**Table 4:** Types of positive outcomes reported by ten or more of 111 respondents.

More energy/alive	24
Clearer thinking	21
Reduced side effects	16
More like myself again	12

**Table 5:** Examples of positive outcomes reported by 111 respondents.

Outcome	Age, Gender, Country
I felt progressively better. More energy, thinking more clearly, more relaxed, more confident, more positive thoughts, more compassion towards other people.	55, M, UK
I began to wake up, my body became less sedated, I felt alive inside again	46, M, Ireland
Clear head immediately. There followed, with my sleeping pattern restored, a vast reduction in hallucinations/talking to myself.	51, M, UK
Self satisfaction - self respect I could express myself - I could cry - I had sexual desire I was no longer a depressed suicidal zombie	40, F, Australia
My emotions came back, I can feel my body again, I can think clearly, my intellect got way better, my interests in life came back.	46, F, Denmark
Much more in touch with emotions and my creative side	51, M, New Zealand
Feeling much more like myself, and free of the side effects and huge doses I had been given. Able to recover!!	61, F, UK
I got my life back. I was free of symptoms (like creaking, painful joints and muscles in the morning) that I didn't realize were tied to the meds.	61, F, USA
Feeling better and less stiffness, easier talking, not feeling like a zombie anymore.	41, M, Netherlands
I felt much better. I stopped being iatrogenically severely depressed and suicidal. I got in touch with my self again, my feelings, my ability to express myself verbally and to think complex thoughts. I could use my body again, I got my fine motor skills back. I was me again - not without problems, but with problems I as a human can live with.	42, F, UK
I began to wake up. From sleeping 16 h a day I was sleeping less and less. My world went from grey 2D to color 3D. I felt alive, my emotions came back I experienced motivation, I lost weight. I was labeled manic but this was me coming back to life	56, F, Denmark
I felt like 'me' again - it was a wonderful feeling	61, F, UK

**Table 6:** Psychosis reported by ten or more of 80 respondents.

‘Psychosis’/’psychotic’ (including 5 ‘rebound’)	37
Hallucinations/voices	25
Paranoia	20
Delusions	10

**Table 7:** Examples of psychosis reported by 80 respondents.

Relapsed quite swiftly	61, M, UK
Psychotic episode after 6 months	36, M, Australia
Increased paranoia and weird thinking that came on gradually	59, M, UK
Total relapse of the condition and with increased paranoia, anxiety, hearing voices, nightmares and delusions.	35, F, UK
Rebound psychosis	49, F, UK
Getting psychotic again due to overload of dopamine in my brain (known effect of antipsychotics) and no skills to handle my emotions after all those years.	36, F, Netherlands
I became acutely psychotic within weeks and was eventually sectioned after several months of pure hell for myself and my family and friends.	58, F, UK
Came off too quickly & became unwell, delusional	35, F, Australia
Had to restart almost immediately. Issues got too bad and I could not control the hallucinations, delusions, psychosis etc etc.	?, F, UK
If I go too fast get rebound psychosis symptoms	62, F, Sweden

**Table 8:** Examples of mixed effects.

Withdrawal and Psychosis (n = 37)	
Unable to sleep (immediate), anxiety, agitation and paranoia increasing linearly with time since last taking the medication, suicidal ideation	52, M, NZ
Severe insomnia and a very bad psychotic reaction, where I climbed a tree in the middle of the night and threw sticks at passers by. Police were called	27, F, UK
My voices were very loud and constant and I was much more paranoid. I had a headache and a stomach ache and felt very overwhelmed. This lasted for about 2 weeks but then I felt much better.	20, F, USA
Some anxiety but when slowed down rate of discontinuation this went away. When in hospital and was taken off seroquel abruptly this caused psychosis that was not present before	65, F, Canada
**Withdrawal and Good Outcome (n = 34)**	
Felt much better. More energy. Some dizziness.	24, M, USA
I experienced feeling my emotions very strongly, compared to the numbness I felt before. It was quite scary	27, F, Denmark
Severe anxiety - but the disabling side effects 'extra pyramidal' stopped	55, M, UK
Brain zaps, confusion, sweat, unsteadiness, a feeling of success, happiness, peace after the extreme physical withdrawals	46, F, UK

#### 3.6.1 Withdrawal effects

Of the 319 naming one or more withdrawal effects (see Table 2), 130 (40.8%) were classified as severe (see Methods). Table 3 provides some examples.

Twenty-nine respondents spontaneously mentioned how long their withdrawal effects lasted, mostly without specifying whether they were still withdrawing or had come off the drug completely. Nine reported that the withdrawal symptoms lasted only two weeks or less. Nine said their symptoms lasted for between one and six months. Two said for between seven and 12 months, and three for one to two years. The other six were clear that they were still experiencing the symptoms, for 15 months, three years, four years, five years, and ten years. One other just said ‘years’. Table 3 includes some examples of these 29 cases.

#### 3.6.2 Positive outcomes

Table 4 summarises the most commonly reported types of positive outcome. Table 5 gives examples.

#### 3.6.3 Psychosis

Table 6 presents the most commonly reported types of psychosis and Table 7 offers examples. In three of these cases there had been no primary or secondary diagnosis indicative of psychosis.

### 3.7 Informed choice

In a Section entitled ‘When you were *first* prescribed antipsychotic medication’, respondents were asked ‘Did the doctor inform you of any possible side effects?’ (Yes/No) and ‘If Yes, what side effects were mentioned?’. None of the 585 who had tried to come off APs (and none of the larger sample of 832), recall being told anything about withdrawal effects, dependence, withdrawal psychosis, worsening TD symptoms, or the need to reduce gradually.

## 4 Discussion

### 4.1 Withdrawal effects

The main finding from this study is that coming off APs is clearly very often a difficult and protracted process. Nearly three quarters (72.5%) of over 500 people who had tried to come off APs reported withdrawal effects, and about half of those people (52.4%) experienced those effects as ‘severe’. This means that more than a third (38.0%) of the sample experienced severe withdrawal symptoms. Just how hard it can be to get off these drugs is further illustrated by one in four (25.7%) having tried four or more times, and a similar number (24.0%) having taken a year or more to completely withdraw.

The finding that the faster people withdrew the less likely they were to experience withdrawal does not contradict the general recommendation that for most people withdrawal is best undertaken slowly and carefully. It seems probable that the likelihood and severity of withdrawal symptom are strong determinants of how quickly people proceed, with those initially experiencing the strongest withdrawal effects consequently being the most cautious and slowest. The length of time on the drugs predicts both the severity of withdrawal effects and the length of time taken to get off them.

Withdrawal effects being related to duration of treatment is unsurprising. It also makes intuitive sense that self-reported outcome (efficacy of the APs) would be negatively related to withdrawal effects. It is less clear why a positive relationship with the prescriber should predict low probability of withdrawal effects. One hypothesis could be that the relationship led to cooperation and support during withdrawal which might, as a result, have been a more gradual and planned process. Future research could usefully explore that possibility.

The types of withdrawal effects that were reported certainly overlap with the ‘classic’ withdrawal symptoms found with other central nervous system drugs (Chouinard et al., 2017). The three most commonly reported symptoms, insomnia, nervousness and extreme feelings, can all be thought of as being the specific result of unblocking a dopamine system that may have been blocked for months or years.

It should be noted that this unblocking probably also underlies many of the positive outcomes reported, including the most commonly reported grouping of ‘more energy/alive’.

The difficulty separating the negative and positive aspects of the unblocking are nicely illustrated by ‘I experienced feeling my emotions very strongly, compared to the numbness I felt before. It was quite scary’.

### 4.2 Psychosis

Three people seemed to develop psychotic symptoms for the first time during withdrawal. It is not possible, however, to tell how many of the other reports of psychosis during withdrawal were withdrawal psychosis (otherwise known as ‘rebound psychosis’ or ‘dopamine supersensitivity psychosis’) and how many were a return of the condition for which the drugs were originally prescribed (Read et al., 2019). It is worth noting that five respondents spontaneously used the term ‘rebound psychosis’ and another wrote: ‘Getting psychotic again due to overload of dopamine in my brain (known effect of antipsychotics) and no skills to handle my emotions after all those years’.

### 4.3 Current guidelines

Discussion about whether the phenomenon in question is best conceptualised as dependence, withdrawal symptoms, addiction or ‘discontinuation syndrome’ (Davies and Read, 2019, Stea, 2020) seems relatively unimportant given that most professional and government bodies, and drug companies, have ignored the phenomenon completely for decades.

Mental health professionals in the UK tend to rely on the highly regarded National Institute for Health and Care Excellence (NICE). NICE guidelines do not acknowledge the existence of the withdrawal effects of antipsychotics, focussing exclusively on remission of psychosis (Cooper, Grünwald, & Horowitz, 2020). As recently as 2020, NICE rejected repeated requests to include antipsychotics in its guidelines for ‘Medicines associated with dependence or withdrawal symptoms’. The requests came from all four groups in NICE’s own guideline scoping process, as well as the All-Party Parliamentary Group for Prescribed Drug Dependence, the International Institute for Psychiatric Drug Withdrawal (www.iipdw.org) , Mind (the UK’s largest mental health NGO), and even the Royal College of Psychiatrists and two drug companies. The stated reasons for this unfortunate but all too typical stance, and a rebuttal for each reason, has been published (Cooper at al., 2020).

Similarly, Public Health England refused requests to include antipsychotics in its otherwise comprehensive and ground-breaking review of ‘Dependence and withdrawal associated with some prescribed medicines’ (Taylor et al., 2019).

Until recently the UK’s Maudsley ‘Prescribing Guidelines in Psychiatry’ only briefly mentioned rebound or super-sensitivity psychosis, and had one sentence on withdrawal effects: ‘Abrupt withdrawal of oral treatment may also lead to discontinuation symptoms (e.g. headache, nausea, insomnia) in some patients’ (Taylor, Barnes & Young, 2018, p. 28). Note the use of the term ‘discontinuation symptoms’’ (rather than ‘withdrawal symptoms’), a drug company term used for decades to minimize the withdrawal effects of antidepressants (Davies & Read, 2019). The most recent, 14th, edition, however, includes eight informative pages on 'Stopping Antipsychotics', using the more accurate term ‘withdrawal symptoms’ (Taylor, Barnes & Young, 2021), mostly derived from Horowitz, Jauhar, Natesan, Murray, & Taylor (2021).

The American Psychiatric Association (2020) makes no mention of the withdrawal effects of APs in its treatment guidance. The U.K.’s Royal College of Psychiatrists (2021) focuses almost entirely on the return of the ‘illness’ or ‘schizophrenia’ but has recently made an important addition to its website: ‘Antipsychotics are not addictive, but your body does get used to them and stopping suddenly may make you feel physically and/or mentally unwell. So it best to reduce the dose of the medication slowly, giving each reduction a few weeks to take effect’.

'Withdrawal effects' from antipsychotics are mentioned in the 'labels' of some antipsychotics, for example clozapine [https://www.medicines.org.uk/emc/product/4411/smpc#gref] and risperidone [https://www.medicines.org.uk/emc/product/5990/smpc#gref]. Some blatantly minimise the effects, for example the Risperidone label states:

“Acute withdrawal symptoms, including nausea, vomiting, sweating, and insomnia have very rarely been described after abrupt cessation of high doses of antipsychotic medicines (see section 4.8). Recurrence of psychotic symptoms may also occur, and the emergence of involuntary movement disorders (such as akathisia, dystonia and dyskinesia) has been reported.”

For aripiprazole withdrawal symptoms are only described for neonates [https://www.medicines.org.uk/emc/product/7073/smpc#gref]. These drug company labels are approved by the UK’s Medicines and Healthcare products Regulatory Agency (MHRA).

Meanwhile, the UK’s largest mental health NGO, Mind (2021), has a whole section on its website called ‘Withdrawal from antipsychotics’ which clearly lists the withdrawal effects and offers advice on how to come off slowly and safely, including ‘Unfortunately, your doctor or psychiatrist may not support your decision to come off antipsychotics. This may mean they don't offer as much help as you would like. Our page on support for coming off psychiatric drugs has information about other ways to find support’.

In 2019 the first national guidelines to properly address withdrawal from APs was published, by the *German Association for Psychiatry, Psychotherapy and Psychosomatics* Germany, (DGPPN, 2019); too late for millions, but a beacon of light for the future if Germany’s example were to be followed.

### 4.4 Next steps

Due to the decades of denial and minimization by psychiatry and the pharmaceutical industry most of the millions that try to come off these drugs every year will have received no support from their prescribers, who are, understandably, focused only on the return of the psychosis. There is no research on doctors’ ability to tell the difference between classic withdrawal symptoms, withdrawal/rebound psychosis and a genuine return of the original condition.

Research is only now beginning, 70 years after the introduction of antipsychotics in the early 1950 s, into how to get off these drugs safely (Brandt et al., 2022, Larsen-Barr et al., 2018a, Larsen-Barr et al., 2018b, Moncrieff et al., 2019, Moncrieff et al., 2020, Steingard, 2018), and how to stay off them (Larsen-Barr & Seymour, 2021). Two recent papers explain the practicalities of reducing antipsychotics carefully so as to minimise withdrawal effects (Horowitz et al., 2021, Horowitz et al., 2022).

A recent *meta*-analysis of 18 studies concluded:.

“We detected moderate evidence of emerging somatic adverse events after discontinuation of first-generation and second-generation antipsychotics, particularly after discontinuation of longer durations of treatment. Tapered discontinuation can mitigate the risk of emerging somatic adverse events after antipsychotic discontinuation. These findings have implications for the safety of treatment discontinuation and could be used for tailored treatment planning”.

The respondents in the current paper also answered several questions about what helped and did not help their efforts to get off the drugs. This will be the subject of a subsequent paper.

## 5 Limitations

This study used a convenience sample rather than a randomised one. The high rates of withdrawal symptoms may, therefore, be partly the result of the sample being biased towards people who were dissatisfied with the drugs overall and had ‘an axe to grind’. However, about half of the sample (52%) reported that the drugs had ‘reduced the problems for which they were prescribed’, which is greater than the rate of positive outcomes found in most AP drug trials. For example, a review of 38 clinical trials, found that only 41% of the AP recipients were classified as ‘responders’ (Lepping et al., 2011).

Online surveys can result in poorer people being underrepresented because of lack of internet access (although income and education levels were not related to withdrawal effects). People who were particularly distressed or disturbed at the time may be underrepresented, because of difficulty using, or lack of interest in, the internet. Men, and all ethnic groups other than ‘white/Caucasian’ were definitely underrepresented.

Another limitation is that the data was self-reported. Memory for events several years ago is less than perfect. For example, some people may have been told about the risk of withdrawal effects but forgotten that. Some of the withdrawal effects and psychosis may not have been related to coming off the APs. It is also possible that some of the positive outcomes reported may have resulted from life changes.

## 6 Conclusions

Prescribers need to inform themselves about the nature, frequency and intensity of withdrawal effects from APs, including withdrawal psychosis (Cooper, Hanratty, Morant & Moncrieff, 2019). AP recipients are entitled to information and support when exercising their right to withdraw from these drugs. Non-medical mental health professionals also need to educate themselves on these issues and be prepared to engage with their clients to support their decision making and, if the client wishes, their withdrawal (Guy, Davies, & Rizq, 2019). National guidelines, professional bodies’ statements, and drug company information all need to be urgently updated so as to adopt an evidence-based approach, rather than the head in the sand approach that has caused unnecessary suffering for millions of people over the past 70 years.

Conflict of interest.

The author receives royalties for Read, J., & Dillon, J. (eds.) (2013). ‘Models of Madness’ (2nd ed.), Routledge, which includes a chapter on antipsychotic drugs which he co-authored.

## Declaration of Competing Interest

The authors declare that they have no known competing financial interests or personal relationships that could have appeared to influence the work reported in this paper.
